# Protease Inhibitor-Dependent Inhibition of Light-Induced Stomatal Opening

**DOI:** 10.3389/fpls.2021.735328

**Published:** 2021-09-10

**Authors:** Tenghua Wang, Wenxiu Ye, Yin Wang, Maoxing Zhang, Yusuke Aihara, Toshinori Kinoshita

**Affiliations:** ^1^Graduate School of Science, Nagoya University, Nagoya, Japan; ^2^Graduate School of Science, Kyoto University, Kyoto, Japan; ^3^Institute of Transformative Bio-Molecules (WPI-ITbM), Nagoya University, Nagoya, Japan; ^4^School of Agriculture and Biology, Shanghai Jiao Tong University, Shanghai, China; ^5^College of Urban and Environmental Sciences and Key Laboratory for Earth Surface Processes of Ministry of Education, Institute of Ecology, Peking University, Beijing, China; ^6^Department of Horticulture, International Research Centre for Environmental Membrane Biology, Foshan University, Foshan, China

**Keywords:** stomata, chemical biology, protease inhibitor, *Commelina*, Arabidopsis

## Abstract

Stomata in the epidermis of plants play essential roles in the regulation of photosynthesis and transpiration. Stomata open in response to blue light (BL) by phosphorylation-dependent activation of the plasma membrane (PM) H^+^-ATPase in guard cells. Under water stress, the plant hormone abscisic acid (ABA) promotes stomatal closure *via* the ABA-signaling pathway to reduce water loss. We established a chemical screening method to identify compounds that affect stomatal movements in *Commelina benghalensis*. We performed chemical screening using a protease inhibitor (PI) library of 130 inhibitors to identify inhibitors of stomatal movement. We discovered 17 PIs that inhibited light-induced stomatal opening by more than 50%. Further analysis of the top three inhibitors (PI1, PI2, and PI3; inhibitors of ubiquitin-specific protease 1, membrane type-1 matrix metalloproteinase, and matrix metalloproteinase-2, respectively) revealed that these inhibitors suppressed BL-induced phosphorylation of the PM H^+^-ATPase but had no effect on the activity of phototropins or ABA-dependent responses. The results suggest that these PIs suppress BL-induced stomatal opening at least in part by inhibiting PM H^+^-ATPase activity but not the ABA-signaling pathway. The targets of PI1, PI2, and PI3 were predicted by bioinformatics analyses, which provided insight into factors involved in BL-induced stomatal opening.

## Introduction

Stomata, each surrounded by a pair of guard cells, are specialized pores on the surface of leaves. Stomatal pores enable plants to regulate CO_2_ uptake and water loss for photosynthesis and transpiration, respectively (Shimazaki et al., 2007). Stomata are also important routes of infection for plant pathogens (Ye and Murata, 2016; Melotto et al., 2017). Stomatal movements are controlled by diverse stimuli, such as blue light (BL), red light (RL), the phytotoxin fusicoccin (FC), CO_2_, the plant hormone abscisic acid (ABA), and microbial elicitors (Munemasa et al., 2015; Inoue and Kinoshita, 2017; Ye et al., 2020). BL receptor phototropins activate PM H^+^-ATPase by phosphorylating the C-terminal Thr (Kinoshita and Shimazaki, 1999; Kinoshita et al., 2001). Activated PM H^+^-ATPase generates a transmembrane electrochemical gradient and establishes an inside-negative electrical potential that drives the influx of K^+^ into guard cells through inward-rectifying voltage-gated K^+^ channels (Schroeder et al., 1987). The accumulation of K^+^ leads to turgor elevation and stomatal opening (Schroeder et al., 2001; Shimazaki et al., 2007). Several signaling mediators are reported to be involved in the signaling pathway of BL-induced stomatal opening. Protein kinase BLUE LIGHT SIGNALING1 (BLUS1) is phosphorylated by phot1 directly as the primary step in phototropin signaling (Takemiya et al., 2013). The Raf-like protein kinase BLUE LIGHT-DEPENDENT H^+^-ATPASE PHOSPHORYLATION (BHP) binds to BLUS1 and regulates BL-dependent phosphorylation of PM H^+^-ATPase (Hayashi et al., 2017). Type 1 protein phosphatase (PP1) positively mediates BL signaling between phototropin and PM H^+^-ATPase (Takemiya et al., 2006). However, the molecular mechanism of signal transduction for BL-induced stomatal opening is incompletely understood (Inoue and Kinoshita, 2017).

The phytohormone ABA is synthesized under drought-stress conditions and reduces stomatal aperture by two mechanisms. ABA promotes stomatal closure by activating outward-rectifying K^+^ channels and anion channels (Kim et al., 2010; Inoue and Kinoshita, 2017; Saito and Uozumi, 2019). In parallel, ABA inhibits light-induced stomatal opening by inhibiting PM H^+^-ATPase and inward-rectifying K^+^ channels in the PM of guard cells. The ABA receptors, PYR/PYL/RCARs, negatively regulate type-2C protein phosphatases (PP2Cs), which inactivate members of the SRK2/SnRK2 family, including open stomata 1 (OST1) (Ma et al., 2009; Park et al., 2009; Santiago et al., 2009; Cutler et al., 2010). Activation of K^+^_out_ channels requires depolarization of the PM by activation of anion channels meditated by the PYR/PYL/RCAR-PP2Cs-SnRK2s pathway (Negi et al., 2008; Vahisalu et al., 2008; Kim et al., 2010; Joshi-Saha et al., 2011). SnRK2s suppress BL-induced phosphorylation of PM H^+^-ATPase and the activity of inward-rectifying K^+^ channels, inhibiting stomatal opening (Sato et al., 2009; Hayashi et al., 2011; Acharya et al., 2013).

Chemical genetics can provide insight into biological systems at the molecular level. It can overcome the problems of traditional genetic approaches, such as gene essentiality and redundancy in gene families (Dejonghe and Russinova, 2017). Chemical screenings related to stomatal movements and development revealed several aspects of stomatal function and development (Kinoshita et al., 2021). Toh et al. (2018) established a comprehensive chemical screening method using *Commelina benghalensis* as a model plant and identified nine stomatal closing compounds (SCL1–SCL9) that suppress light-induced stomatal opening by >50%, and two compounds (temsirolimus and CP-100356) that induce stomatal opening in the dark. This indicates the feasibility of chemical approaches for analyzing stomatal movements. Notably, BHP, a factor involved in BL-induced stomatal opening, was identified by a combination of chemical screening focused on BL-induced phosphorylation of PM H^+^-ATPase in guard cells and reverse genetics in *Arabidopsis thaliana* (Hayashi et al., 2017). In this study, we performed chemical genetics screening of a protease inhibitor (PI) library and identified three inhibitors of light-induced stomatal opening and BL-induced phosphorylation of PM H^+^-ATPase in guard cells. Interestingly, spraying leaves onto monocot and dicot plants with PI1 suppressed wilting of leaves, indicating that inhibition of stomatal opening by PI1 decreases water loss in plants. To our knowledge, screening of PIs that affect stomatal movements has not been reported. We also investigated the molecular mechanisms of these inhibitors and laid the foundation for the identification of novel factors in the BL-signaling pathway.

## Materials and Methods

### Plant Materials and Growth Conditions

Plants of *Commelina benghalensis* ssp. were cultured in soil at 25 ± 3°C in a greenhouse for 4 weeks. *Arabidopsis thaliana* (ecotype Col-0) plants were grown in soil for 4–6 weeks under controlled conditions (20–24°C, 55–70% humidity, 16 h light/8 h dark) in growth chambers. Seeds were vernalized at 4°C in the dark for 2 days before being transferred to soil. Oat (*Avena sativa*) seedlings were grown in growth chambers with same conditions of Arabidopsis for 10 days.

### Chemicals

A protease inhibitor (PI) library (130 PIs dissolved in DMSO at 10 mM) was purchased from APExBIO Company. Repurchased chemicals were SJB3-019A (CAS No. 2070015-29-9, >99.00% purity, Medchem Express), NSC 405020 (CAS No. 7497-07-6, >99.07% purity, Sellek Chemicals), SB-3CT (CAS No. 292605-14-2, >98.00% purity, TCI). All chemicals were stored at −20°C.

### Chemical Screening

Screening of chemicals was performed as described previously (Toh et al., 2018) with some modifications. Four-week-old *C*. *benghalensis* plants were transferred from a greenhouse to a dark room for incubation overnight to ensure complete stomatal closure on day 2. Leaf discs of 4 mm diameter were excised from dark-adapted *C*. *benghalensis* using a hole punch under dim light. The leaf discs were floated on basal reaction buffer [5 mM MES-BTP (Bis-trispropane), pH 6.5, 50 mM KCl and 0.1 mM CaCl_2_] with chemical compounds and incubated in light (150 μmol m^–2^ s^–1^ red light and 50 μmol m^–2^ s^–1^ BL) or in the dark for 3 h. Leaf discs were incubated with compounds for 30 min before light exposure. Stomatal apertures of the abaxial epidermis were measured as described previously (Toda et al., 2018). Inhibition by PI1, PI2, and PI3 of stomatal opening was identified. To assay viability, abaxial epidermis was removed from leaf discs using forceps after chemical and light treatments and incubated in fresh basal reaction buffer containing 1 μg/mL fluorescein diacetate (FDA) for 30 min in the dark. Epidermis was washed three times with Milli-Q water to remove FDA. Fluorescence microscopy was used to detect fluorescence.

### Immunohistochemical Analysis

Phosphorylation of PM H^+^-ATPase was determined by immunohistochemical analysis following previous methods using ImageJ (Hayashi et al., 2011; Ando and Kinoshita, 2018).

### Detection of Phototropin Autophosphorylation

Leaf discs excised from dark-adapted 5-week-old Arabidopsis were incubated in basal reaction buffer with PIs (100 μM) or an equal volume of DMSO in the dark for 1 h in advance. Leaf discs were irradiated with BL (50 μmol m^–2^ s^–1^) or kept in the dark for 1 h. Immunoblot analysis was performed as described previously (Kinoshita et al., 2003; Inoue et al., 2008). Proteins were separated by 7.5% sodium dodecyl sulfate-polyacrylamide gel electrophoresis (SDS-PAGE) and transferred to nitrocellulose membranes. The primary and secondary antibodies were anti-phot1 (Emi et al., 2005; Inoue et al., 2008) and anti-rabbit IgG HRP (Bio-Rad, CA) respectively.

### Quantification of ABA-Dependent Gene Expression and Germination Assay

We determined the expression of the ABA-maker genes *RAB18* (At5g66400) and *RD29B* (At5g52300) by quantitative RT-PCR in Arabidopsis (Tomiyama et al., 2014). Two-week-old seedlings cultured in solid 1/2 MS medium were transferred to liquid 1/2 MS medium containing 50 μM ABA or 100 μM PIs or an equal volume of DMSO and irradiated with white fluorescent light for 3 h at 24°C. Total RNA was isolated from seedlings and first-strand cDNA was synthesized using the Prime Script II First Strand cDNA Synthesis Kits (TaKaRa, Tokyo, Japan). Quantitative RT-PCR was performed using Power SYBR Green PCR Master Mix and the Step One Real-Time PCR System (Applied Biosystems, Foster City, CA). *RAB18*, *RD29B*, and *TUB2* cDNAs were amplified using specific primers (Supplementary Table 1).

Arabidopsis seeds were incubated in MQ water containing 50 μM ABA or 100 μM PIs or an equal volume of DMSO in 96-well plates sealed with surgical tapes. The space between wells was filled with water. The plates were kept at 4°C in darkness for 2 days and transferred to an illumination incubator at 24°C with a 16 h light/8 h dark cycle for 7 days.

### Chemical Spraying for Drought Tolerance Assay

Drought tolerance assays were performed as described previously (Toh et al., 2018) with slight modifications. Rose bouquets purchased from a local shop were cultured in a light incubator for 2 days for environmental adaption. Rose leaves and 10-day-old oat seedlings were sprayed with 100 μM PIs or an equal volume of DMSO in 0.05% Approach BI (Maruwa Biochemical) and 0.02% Silwet L77 (Biomedical Science) under white light (50 μmol m^–2^ s^–1^) and 70% humidity for 3 h at 24°C. Rose and oat leaves were removed and illuminated with 50 μmol m^–2^ s^–1^ white light for 8 h and 30 min, respectively, at 27°C with 40% humidity.

## Results

### Chemical Screening for Stomatal Movement

We evaluated the ability of 130 PIs on stomatal movements (Supplementary Figure 1). *C*. *benghalensis* is useful for determination of stomatal aperture due to its larger stomata compared to *A. thaliana*. Seventeen inhibitors suppressed light-induced stomatal opening by >50% compared to the control (Supplementary Figure 2). No PI induced stomatal opening in the dark (Supplementary Figure 3). Staining with the fluorescent dye FDA showed that none of the PIs affected cell viability (Supplementary Figure 4).

**FIGURE 1:**
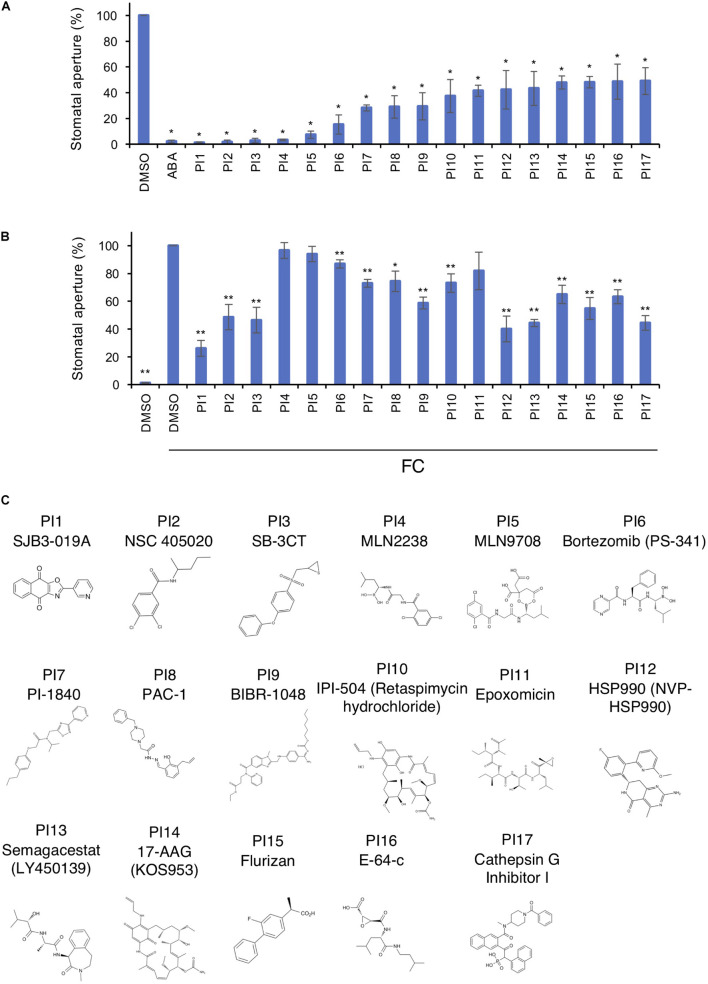
Inhibition of light- and FC-induced stomatal opening by proteinase inhibitors (PIs). **(A)** Inhibition by PIs of light-induced stomatal opening. Leaf discs were pre-treated with compounds for 30 min in the dark and illuminated with light (150 μmol m^–2^ s^–1^ red light and 50 μmol m^–2^ s^–1^ BL) for 3 h. ABA, 10 μM; PIs, 100 μM. Means ± SE (*n* = 5, 30 stomata per 2 leaf discs per repeat). Student’s *t*-test, *P < 0.01. **(B)** Suppression by PIs of FC-induced stomatal opening. Leaf discs were incubated with PIs in basal buffer for 30 min, FC was added, and incubated for 3 h in the dark. PIs, 100 μM; FC, 10 μM. Means ± SE (*n* = 4, >50 stomata from 3 leaf discs per repeat). Student’s *t*-test, *P < 0.05, **P < 0.01. **(C)** Chemical structures of PIs.

Figure 1A shows inhibition of light-induced stomatal opening by 17 chemicals selected through screenings. These inhibitors were designated PI1–PI17 and their structures are shown in Figure 1C. The targets of PIs in mammals are shown in Supplementary Table 2. The fungal phytotoxin FC leads to irreversible stomatal opening in the dark by binding of 14-3-3 protein to phosphorylated PM H^+^-ATPase, preventing dephosphorylation of PM H^+^-ATPase (Kinoshita and Shimazaki, 2001). To investigate whether the 17 PIs function upstream or downstream of PM H^+^-ATPase activation, we examined their effects on FC-induced stomatal opening (Figure 1B). PI4, PI5 and PI11 did not inhibit FC-induced stomatal opening, indicating that they may have effects upstream of PM H^+^-ATPase. The other PIs significantly inhibited stomatal opening induced by FC to varying degrees, suggesting that they regulate downstream of PM H^+^-ATPase, but not the BHP or PP1, or inhibit PM H^+^-ATPase phosphorylation.

**FIGURE 2:**
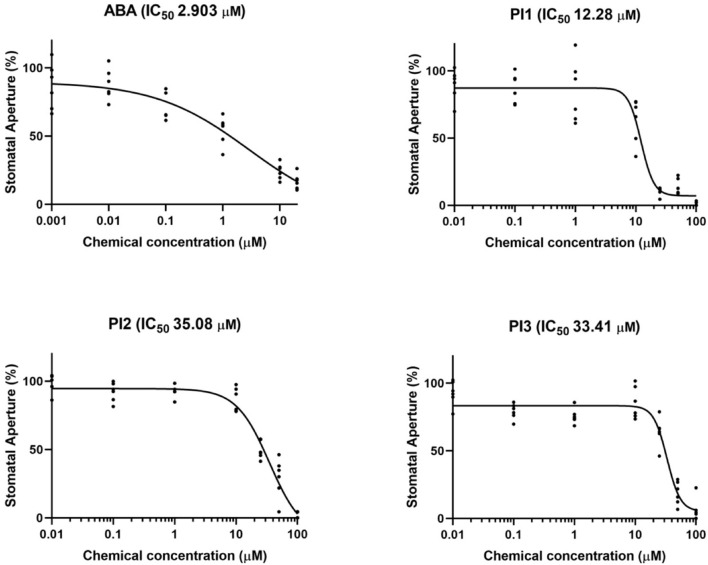
IC_50_ curves of ABA, PI1, PI2, and PI3 for light-induced stomatal opening. The concentrations used to obtain IC_50_ curves were 0.001, 0.01, 0.1, 1, 10, and 20 μM for ABA and 0.01, 0.1, 1, 10, 25, 50, and 100 μM for PIs. IC_50_ values were computed by GraphPad Prism (*n* = 6, 30 stomata from 3 leaf discs per concentration).

Next, we examined the concentration-dependency of the top three inhibitors (PI1, an inhibitor of ubiquitin-specific protease 1; PI2 and PI3, inhibitors of membrane type-1 matrix metalloproteinase and matrix metalloproteinase-2, respectively) for light-induced stomatal opening (Figure 2). Leaf discs were treated with the indicated compounds and exposed to light for 3 h. The 50% inhibitory concentration (IC_50_) values of PI1, PI2, and PI3 were 12.28, 35.08, and 33.41 μM, respectively, indicating that PI1 inhibited light-induced stomatal opening more efficiently than PI2 and PI3 at low concentrations. The IC_50_ value of ABA was 2.903 μM.

### Effects of PI1, PI2, and PI3 on Phosphorylation of PM H^+^-ATPase and Phototropin

**FIGURE 3:**
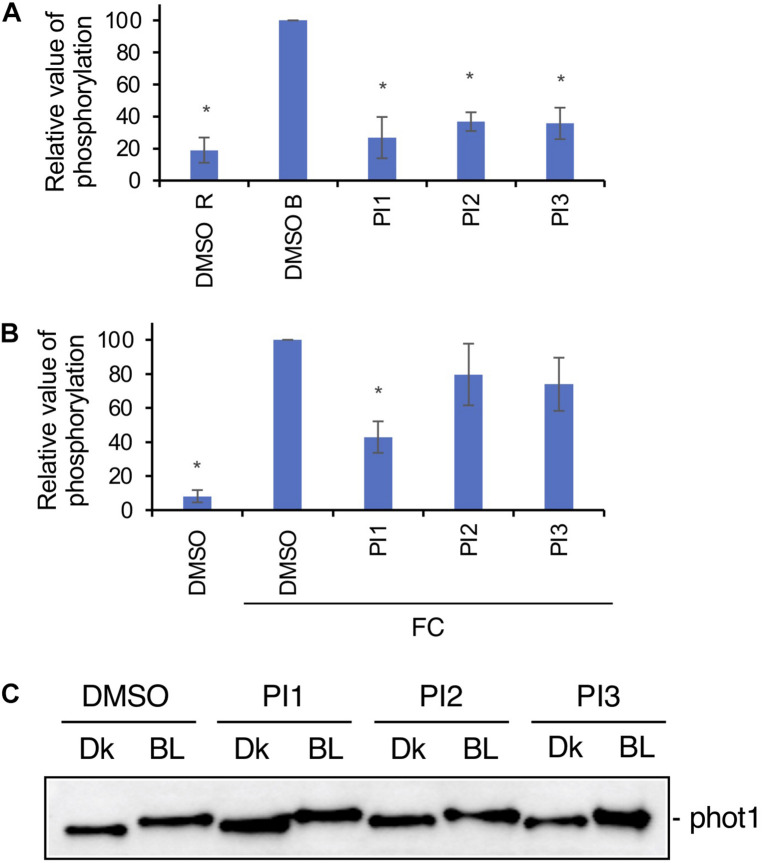
Effects of PIs on phosphorylation of PM H^+^-ATPase in guard cells and phototropin. **(A)** Inhibition by PIs of BL-dependent H^+^-ATPase phosphorylation in guard cells of Arabidopsis. Leaf epidermis was pre-treated with compounds for 20 min in the dark and illuminated with 50 μmol m^–2^ s^–1^ red light for 20 min (DMSO R), after which 10 μmol m^–2^ s^–1^ BL was superimposed on the red light for 2.5 min (DMSO B, PI1, PI2, PI3). PIs, 100 μM. Means ± SE (*n* = 3). Student’s *t*-test, *P < 0.01. **(B)** Suppression by PIs of FC-induced phosphorylation of PM H^+^-ATPase. Leaf epidermis from Arabidopsis was incubated with DMSO or PIs in basal buffer for 30 min in the dark, FC was added, and incubated for 30 min under red light (50 μmol m^–2^ s^–1^). PIs, 100 μM; FC, 10 μM. Means ± SE (*n* = 3). Student’s *t*-test, *P < 0.01. Phosphorylation of PM H^+^-ATPase was detected using an anti-pThr (phosphorylated threonine, the penultimate residue of the PM H^+^-ATPase) primary antibody and Alexa 488 conjugated anti-rabbit IgG secondary antibody in panels **(A,B)**. **(C)** Effects of PI1, PI2, and PI3 on BL-induced phot1 autophosphorylation in Arabidopsis detected by mobility shift assay. Dk, leaf discs from Arabidopsis treated with DMSO or PIs and incubated in the dark for 2 h. BL, leaf discs treated with DMSO or PIs, incubated in the dark for 1 h and transferred to BL (50 μmol m^–2^ s^–1^) for 1 h. PIs, 100 μM. Phot1 protein was detected using an anti-phot1 antibody. Experiments were repeated on three different occasions with similar results.

To investigate whether PI1, PI2, and PI3 influence BL-dependent phosphorylation of PM H^+^-ATPase, we performed immunohistochemical analysis of guard cells using epidermis isolated from *Arabidopsis thaliana* (Hayashi et al., 2011). BL-induced phosphorylation was suppressed completely by these three PIs (Figure 3A). We next determined the inhibitory effects of PI1, PI2, and PI3 on FC-induced phosphorylation of the PM H^+^-ATPase. FC-induced phosphorylation was significantly inhibited by PI1, and partially by PI2 and PI3 (Figure 3B). The different inhibitory effects of these inhibitors are consistent with their IC_50_ values for light-induced stomatal opening (Figure 2).

Next, we determined whether PI1, PI2, and PI3 inhibit the kinase activity of phototropin. Autophosphorylation of phot1 induced by BL in leaves was detected by mobility shift assay using an antibody against phot1 in Western blotting (Emi et al., 2005; Inoue et al., 2008). PI1, PI2, and PI3 did not affect the BL-induced mobility shift of phot1 (Figure 3C), suggesting that PI1, PI2, and PI3 inhibit light-induced stomatal opening without affecting phototropin activity.

### Effects of PIs on ABA-Responsive Genes and Germination

**FIGURE 4:**
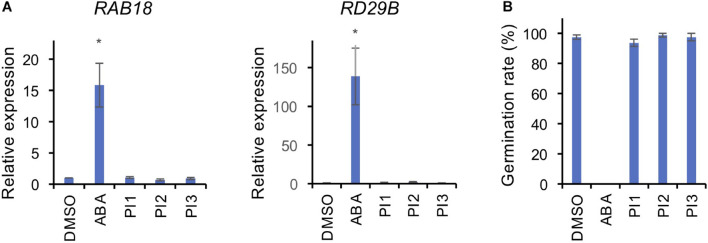
Effects of PIs on ABA-induced gene expression and seed germination. **(A)** Expression levels of ABA-responsive genes, *RAB18* and *RD29B*, were determined by qRT-PCR in response to ABA and PIs in Arabidopsis seedlings. *TUB2* was used as the internal standard. Two-week-old seedlings were treated with 50 μM ABA or 100 μM PIs or an equal volume of DMSO under white light for 3 h at 24°C. Means ± SE (*n* = 3; 3 whole seedlings per replicate). Student’s *t*-test, *P < 0.05. **(B)** Effects of PIs on Arabidopsis seed germination. Seeds were immersed in water with 50 μM ABA or 100 μM PIs or an equal volume of DMSO in the dark for 2 days for vernalization, and 7 days for germination under light. Means ± SE (*n* = 4; 20 seeds per replicate).

To investigate whether PI1, PI2, and PI3 inhibit BL-induced stomatal opening in a manner similar to ABA, we analyzed the expression levels of the ABA-responsive genes, *RAB18* and *RD29B*, by quantitative RT-PCR in Arabidopsis seedlings and germination of Arabidopsis seeds (Toh et al., 2018). Incubation of seedlings with 50 μM ABA for 3 h induced expression of *RAB18* and *RD29B* (Figure 4A). By contrast, PI1, PI2, and PI3 did not induce the expression of ABA-responsive genes (Figure 4A). Moreover, ABA at 50 μM inhibited germination of Arabidopsis seeds (Figure 4B). However, PI1, PI2, and PI3 had no effect on germination (Figure 4B). The results suggest that the mechanisms underlying the inhibitory effects of the PIs and ABA on light-induced stomatal opening are different and that the PIs have more specific roles in regulating stomatal movement.

### PI1 Reduces Water Loss in Plants

**FIGURE 5:**
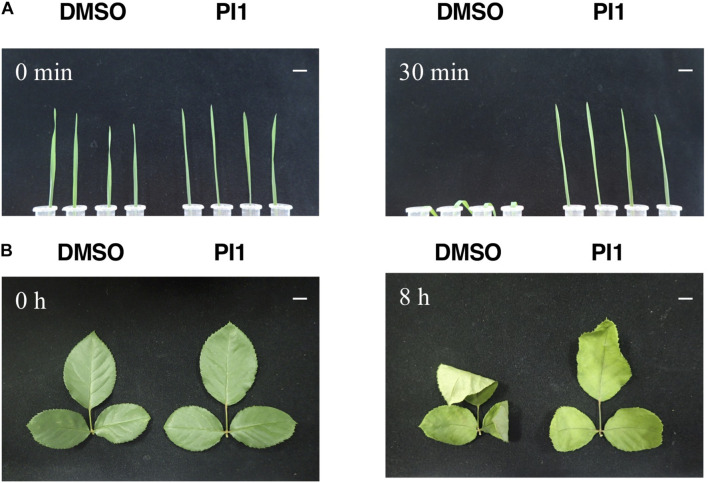
Effect of PI1 on leaf wilting. Oat leaves **(A)** and rose leaves **(B)**. Ten-day-old oat seedlings grown in soil **(A)** and rose leaves in bouquets **(B)** were sprayed with 100 μM PI1 or an equal volume of DMSO in 0.05% Approach BI and 0.02% Silwet L77 and incubated under white light (50 μmol m^–2^ s^–1^) and 70% relative humidity at 24°C for 3 h. Next, leaves were excised and incubated for 30 min (oat) and 8 h (rose) at 27°C under 50 μmol m^–2^ s^–1^ at ∼40% relative humidity. Scale bars = 1 cm. Experiments were repeated on three different occasions with similar results.

SCL1, which reportedly induces stomatal closure, prevents wilting of detached oat and rose leaves, which is likely to be by inhibiting light-induced stomatal opening (Toh et al., 2018). We investigated the effect of PI1 on drought resistance using the same method with slight modifications. Oat and rose leaves were sprayed with PI1 at 100 μM and incubated for 3 h, and the leaves were detached. PI1 inhibited leaf withering in oat plants compared with the control (Figure 5A). Rose leaves treated with PI1 wilted slower than that of control (Figure 5B). Therefore, PI1 improves drought resistance in dicot and monocot plants.

## Discussion

### Effect of PI1 on Light-Induced Stomatal Opening

**FIGURE 6:**
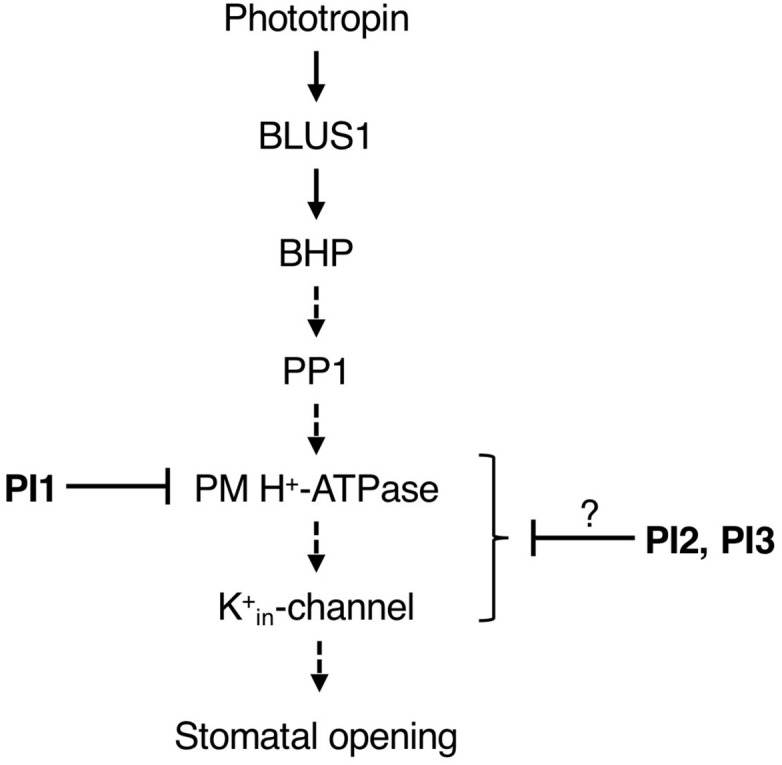
Proposed model of the inhibitory effects of PI1, PI2, and PI3 on the signaling pathway of BL-dependent stomatal opening. The pathway starts from phototropin, followed by BLUS1, BHP, PP1, a key enzyme PM H^+^-ATPase and K^+^_in_-channel. PI1, PI2, and PI3 inhibited BL-induced phosphorylation of PM H^+^-ATPase. Arrows indicate positive regulation. Blunt arrows indicate repression.

We screened for compounds that affect light-induced stomatal opening using a commercially available PI library (APExBIO) and identified 17 PIs that significantly suppressed light-induced stomatal opening by >50% (Figure 1A). PI1, which had the greatest inhibitory effect on stomatal opening, inhibited both light- and FC-induced stomatal opening (Figures 1A,B) and both BL- and FC-induced phosphorylation of PM H^+^-ATPase in guard cells (Figures 3A,B). By contrast, PI1 had no effect on phototropin activity (Figure 3C) and did not induce ABA-dependent responses, such as inhibition of seed germination and expression of ABA-responsive genes (Figure 4). These results suggest that PI1-sensitive proteases mediate phosphorylation of PM H^+^-ATPase (Figure 6) by positively regulating unidentified kinases that directly phosphorylate PM H^+^-ATPase or down-regulating protein phosphatases that directly dephosphorylate PM H^+^-ATPase (Inoue and Kinoshita, 2017). PI2 and PI3 markedly suppressed light-induced stomatal opening (Figure 1A), and partially inhibited FC-induced stomatal opening (Figure 1B) and FC-induced phosphorylation of PM H^+^-ATPase in guard cells (Figure 3B). Therefore, the targets of PI2 and PI3 are likely to include PM H^+^-ATPase and downstream components of light-induced stomatal opening (Figure 6).

It is worth noting that PI4, PI5 and PI11 suppressed light-induced stomatal opening and didn’t inhibit FC-induced stomatal opening significantly (Figures 1A, B), suggesting that the targets of these inhibitors are components upstream of BL-signaling pathway, such as phototropins, BLUS1, BHP, and PP1.

### Candidate Targets of PI1 in Arabidopsis

In mammalian cells, PI1 is a specific inhibitor of ubiquitin-specific protease 1 (USP1) (Mistry et al., 2013; Das et al., 2017). To predict the target of PI1 in Arabidopsis, we performed a BLAST search (The National Center for Biotechnology Information)^1^ using the amino acid sequence of USP1 from *Homo sapiens* and found two ubiquitin specific processing proteases (UBP3 and UBP4) with relatively high identity (around 33%) to USP1 (Supplementary Table 3). The UBP family in Arabidopsis has a similar sequence to human USPs and plays a critical role in de-ubiquitination of proteins (March and Farrona, 2018). In Arabidopsis, the UBP family has 27 members (Supplementary Figure 5A), which are divided into 14 subfamilies based on their domains (Yan et al., 2000; Liu et al., 2008; Zhou et al., 2017). All Arabidopsis UBPs contain a UBP domain, and each subfamily shares other conserved domains that are likely to mediate protein-protein interactions (Liu et al., 2008; Komander et al., 2009; March and Farrona, 2018). To our knowledge, the binding site of USP1 with PI1 is unknown.

We hypothesized that some of the 27 UBPs participate in the stomatal-opening pathway in response to BL. These UBPs may function separately or redundantly and up-regulate the unidentified kinases that directly phosphorylate or down-regulate phosphatases that dephosphorylate the PM H^+^-ATPase. We also investigated their expression levels in guard cells using Arabidopsis eFP Browser^2^ (Supplementary Figure 5B). *UBP6* and *UBP13* exhibited the highest expression levels. Stomatal closure in the mutant of *ubp24* was less sensitive to ABA than the WT and the stomata of complemented transgenic plants showed sensitivity to ABA similar to the WT, indicating that UBP24 is involved in ABA-mediated stomatal closure (Zhao et al., 2016). However, PI1 is less likely to modulate the factors in the ABA signaling pathway (Figure 4), so UBP24 may not be the target of PI1.

### Presumption of PI2 and PI3 Targets in Arabidopsis

In mammals, the targets of PI2 and PI3 are membrane type-1 matrix metalloproteinase (MT1-MMP/MMP-14) and matrix metalloproteinase-2 (MMP-2), respectively (Kleifeld et al., 2001; Remacle et al., 2012). MMPs are a large family of zinc- and calcium-dependent endopeptidases (Rawlings et al., 2006). In human, MT1-MMP and MMP-2 have a common domain structure that includes a signal peptide, a propeptide, a catalytic domain with the active zinc binding site, a hinge region, and a hemopexin-like domain. MT1-MMP has two domains absent in MMP-2—membrane linker and cytoplasmic domains (Overall and López-Otín, 2002). PI2 directly targets the hemopexin-like domain of MT1-MMP (Remacle et al., 2012). PI3 is an MMP-2 inhibitor that specifically binds the catalytic zinc ion (Kleifeld et al., 2001). In Arabidopsis, five genes named *At1-MMP* to *At5-MMP* encode MMPs with common structural features with mammalian MMPs but lacking a hemopexin-like domain (Maidment et al., 1999; Marino and Funk, 2012; Supplementary Figures 6A,B). We evaluated the expression levels of five MMPs in Arabidopsis guard cells using an eFP Browser; the expression of *AT5-MMP* was highest (Supplementary Figure 6C). The identities of At-MMPs aligned with human MMP-2 are shown in Supplementary Table 3. In soybean, glycine max1-matrix metalloproteinase (GM1-MMP) and glycine max2-matrix metalloproteinase (GM2-MMP) are PM proteins, and the stomatal aperture of transgenic Arabidopsis plants overexpressing *GM1-MMP* and *GM2-MMP* was significantly larger than the WT (Liu et al., 2017, 2018), suggesting that *GM1-MMP* and *GM2-MMP* regulate stomatal opening. Therefore, *AtMMPs* are candidate targets of PI3 in Arabidopsis.

## Conclusion

In conclusion, we identified seventeen PIs that suppress stomatal opening under light and found that among them, PI1, PI2, and PI3 inhibit phosphorylation of penultimate residue, threonine, in guard-cell PM H^+^-ATPase, which is important for stomatal opening. Our findings will facilitate identification of novel regulators and provide insight into the molecular mechanisms of light-induced stomatal opening. Further investigations of these inhibitors are expected to shed light on the BL- signaling pathway and future development of agrochemicals that control drought tolerance and plant growth. In particular, drought stress severely affects quality and yield of crops. If agrochemicals confer drought tolerance like the compounds identified in this study, it is possible not only to improve the yield of crops but also to cultivate crops in non-farming areas due to a shortage of rainfall.

## Data Availability Statement

The raw data supporting the conclusions of this article will be made available by the authors, without undue reservation.

## Author Contributions

TW, WY, and TK designed the experiments and wrote the manuscript. TW, WY, YW, MZ, YA, and TK performed the experiments. All authors reviewed the manuscript.

## Conflict of Interest

The authors declare that the research was conducted in the absence of any commercial or financial relationships that could be construed as a potential conflict of interest.

## Publisher’s Note

All claims expressed in this article are solely those of the authors and do not necessarily represent those of their affiliated organizations, or those of the publisher, the editors and the reviewers. Any product that may be evaluated in this article, or claim that may be made by its manufacturer, is not guaranteed or endorsed by the publisher.

## Footnotes

1
https://www.ncbi.nlm.nih.gov/

2
http://bar.utoronto.ca/efp/cgi-bin/efpWeb.cgi?dataSource=Guard_Cell

## References

[B1] Acharya B. R. Jeon B. W. Zhang W. Assmann S. M. (2013). Open Stomata 1 (OST1) is limiting in abscisic acid responses of *Arabidopsis* guard cells. *New Phytol*. 200 1049–1063. 10.1111/nph.12469 24033256

[B2] Ando E. Kinoshita T. (2018). Red light-induced phosphorylation of plasma membrane H^+^-ATPase in stomatal guard cells. *Plant Physiol*. 178 838–849. 10.1104/pp.18.00544 30104254 PMC6181031

[B3] Cutler S. R. Rodriguez P. L. Finkelstein R. R. Abrams S. R. (2010). Abscisic acid: emergence of a core signaling network. *Annu. Rev. Plant Biol.* 61 651–679. 10.1146/annurev-arplant-042809-112122 20192755

[B4] Das D. S. Das A. Ray A. Song Y. Samur M. K. Munshi N. C. (2017). Blockade of deubiquitylating enzyme USP1 inhibits DNA repair and triggers apoptosis in multiple myeloma cells. *Clin. Cancer Res.* 23 4280–4289. 10.1158/1078-0432.CCR-16-2692 28270494 PMC5540781

[B5] Dejonghe W. Russinova E. (2017). Plant chemical genetics: from phenotype-based screens to synthetic biology. *Plant Physiol*. 174 5–20. 10.1104/pp.16.01805 28275150 PMC5411137

[B6] Emi T. Knoshita T. Sakamoto K. Mineyuki Y. Shimazaki K.-I. (2005). Isolation of a protein interacting with Vfphot1a in guard cells of *Vicia faba*. *Plant Physiol*. 138 1615–1626. 10.1104/pp.104.052639 15980204 PMC1176431

[B7] Hayashi M. Inoue S. Takahashi K. Kinoshita T. (2011). Immunohistochemical detection of blue light-induced phosphorylation of the plasma membrane H^+^-ATPase in stomatal guard cells. *Plant Cell Physiol*. 52 1238–1248. 10.1093/pcp/pcr072 21666226

[B8] Hayashi M. Inoue S. Ueno Y. Kinoshita T. (2017). A Raf-like protein kinase BHP mediates blue light-dependent stomatal opening. *Sci. Rep.* 7:45586. 10.1038/srep45586 28358053 PMC5372365

[B9] Inoue S. Kinoshita T. (2017). Blue light regulation of stomatal opening and the plasma membrane H^+^-ATPase. *Plant Physiol*. 174 531–538. 10.1104/pp.17.00166 28465463 PMC5462062

[B10] Inoue S. Kinoshita T. Matsumoto M. Nakayama K. I. Doi M. Shimazaki K. (2008). Blue light-induced autophosphorylation of phototropin is a primary step for signaling. *Proc. Natl. Acad. Sci. U.S.A.* 105 5626–5631. 10.1073/pnas.0709189105 18378899 PMC2291087

[B11] Joshi-Saha A. Valon C. Leung J. (2011). Abscisic acid signal off the STARting block. *Mol. Plant* 4 562–580. 10.1093/mp/ssr055 21746700

[B12] Kim T. H. Bohmer M. Hu H. Nishimura N. Schroeder J. I. (2010). Guard cell signal transduction network: advances in understanding abscisic acid, CO_2_, and Ca^2+^ signaling. *Annu. Rev. Plant Biol.* 61 561–591. 10.1146/annurev-arplant-042809-112226 20192751 PMC3056615

[B13] Kinoshita T. Doi M. Suetsugu N. Kagawa T. Wada M. Shimazaki K. (2001). phot1 and phot2 mediate blue light regulation of stomatal opening. *Nature* 414 656–660. 10.1038/414656a 11740564

[B14] Kinoshita T. Emi T. Tominaga M. Sakamoto K. Shigenaga A. Doi M. (2003). Blue light- and phosphorylation-dependent binding of a 14-3-3 protein to phototropins in stomatal guard cells of broad bean. *Plant Physiol*. 133 1453–1463. 10.1104/pp.103.029629 14605223 PMC300702

[B15] Kinoshita T. Shimazaki K. I. (1999). Blue light activates the plasma membrane H^+^-ATPase by phosphorylation of the C-terminus in stomatal guard cells. *EMBO J*. 18 5548–5558. 10.1093/emboj/18.20.5548 10523299 PMC1171623

[B16] Kinoshita T. Shimazaki K. I. (2001). Analysis of the phosphorylation level in guard-cell plasma membrane H^+^-ATPase in response to fusicoccin. *Plant Cell Physiol*. 42 424–432. 10.1093/pcp/pce055 11333314

[B17] Kinoshita T. Toh S. Torii K. U. (2021). Chemical control of stomatal function and development. *Curr. Opin. Plant Biol.* 60:102010. 10.1016/j.pbi.2021.102010 33667824

[B18] Kleifeld O. Kotra L. P. Gervasi D. C. Brown S. Bernardo M. M. Fridman R. (2001). X-ray absorption studies of human matrix metalloproteinase-2 (MMP-2) bound to a highly selective mechanism-based inhibitor. Comparison with the latent and active forms of the enzyme. *J. Biol. Chem.* 276 17125–17131. 10.1074/jbc.M011604200 11278946

[B19] Komander D. Clague M. J. Urbé S. (2009). Breaking the chains: structure and function of the deubiquitinases. *Nat. Rev. Mol. Cell Biol.* 10 550–563. 10.1038/nrm2731 19626045

[B20] Liu S. Jia Y. Zhu Y. Zhou Y. Shen Y. Wei J. (2018). Soybean matrix metalloproteinase *Gm2-MMP* relates to growth and development and confers enhanced tolerance to high temperature and humidity stress in transgenic *Arabidopsis*. *Plant Mol. Biol. Rep.* 36 94–106. 10.1007/s11105-017-1065-8

[B21] Liu S. Liu Y. Jia Y. Wei J. Wang S. Liu X. (2017). Gm1-*MMP* is involved in growth and development of leaf and seed, and enhances tolerance to high temperature and humidity stress in transgenic *Arabidopsis*. *Plant Sci*. 259 48–61. 10.1016/j.plantsci.2017.03.005 28483053

[B22] Liu Y. Wang F. Zhang H. He H. Ma L. Deng X. W. (2008). Functional characterization of the *Arabidopsis ubiquitin-specific protease* gene family reveals specific role and redundancy of individual members in development. *Plant J*. 55 844–856. 10.1111/j.1365-313X.2008.03557.x 18485060

[B23] Ma Y. Szostkiewicz I. Korte A. Moes D. Yang Y. Christmann A. (2009). Regulators of PP2C phosphatase activity function as abscisic acid sensors. *Science* 324 1064–1068. 10.1126/science.1172408 19407143

[B24] Maidment J. M. Moore D. Murphy G. P. Murphy G. Clark I. M. (1999). Matrix metalloproteinase homologues from *Arabidopsis thaliana.* Expression and activity. *J. Biol. Chem.* 274 34706–34710. 10.1074/jbc.274.49.34706 10574937

[B25] March E. Farrona S. (2018). Plant deubiquitinases and their role in the control of gene expression through modification of histones. *Front. Plant Sci.* 8:2274. 10.3389/fpls.2017.02274 29387079 PMC5776116

[B26] Marino G. Funk C. (2012). Matrix metalloproteinases in plants: a brief overview. *Physiol. Plant.* 145 196–202. 10.1111/j.1399-3054.2011.01544.x 22084906

[B27] Melotto M. Zhang L. Oblessuc P. R. He S. Y. (2017). Stomatal defense a decade later. *Plant Physiol*. 174 561–571. 10.1104/pp.16.01853 28341769 PMC5462020

[B28] Mistry H. Hsieh G. Buhrlage S. J. Huang M. Park E. Cuny G. D. (2013). Small-molecule inhibitors of USP1 target ID1 degradation in leukemic cells. *Mol. Cancer Ther.* 12 2651–2662. 10.1158/1535-7163.MCT-13-0103-T 24130053 PMC4089878

[B29] Munemasa S. Hauser F. Park J. Waadt R. Brandt B. Schroeder J. I. (2015). Mechanisms of abscisic acid-mediated control of stomatal aperture. *Curr. Opin. Plant Biol*. 28 154–162. 10.1016/j.pbi.2015.10.010 26599955 PMC4679528

[B30] Negi J. Matsuda O. Nagasawa T. Oba Y. Takahashi H. Kawai-Yamada M. (2008). CO_2_ regulator SLAC1 and its homologues are essential for anion homeostasis in plant cells. *Nature* 452 483–486. 10.1038/nature06720 18305482

[B31] Overall C. M. López-Otín C. (2002). Strategies for MMP inhibition in cancer: innovations for the post-trial era. *Nature Rev. Cancer* 2 657–672. 10.1038/nrc884 12209155

[B32] Park S. Y. Fung P. Nishimura N. Jensen D. R. Fujii H. Zhao Y. (2009). Abscisic acid inhibits type 2C protein phosphatases via the PYR/PYL family of START proteins. *Science* 324 1068–1071. 10.1126/science.1173041 19407142 PMC2827199

[B33] Rawlings N. D. Morton F. R. Barrett A. J. (2006). MEROPS: the peptidase database. *Nucleic Acids Res*. 34 D270–D272. 10.1093/nar/gkp971 16381862 PMC1347452

[B34] Remacle A. G. Golubkov V. S. Shiryaev S. A. Dahl R. Stebbins J. L. Chernov A. V. (2012). Novel MT1-MMP small-molecule inhibitors based on insights into hemopexin domain function in tumor growth. *Cancer Res*. 72 2339–2349. 10.1158/0008-5472.CAN-11-4149 22406620 PMC3342448

[B35] Saito S. Uozumi N. (2019). Guard cell membrane anion transport systems and their regulatory components: an elaborate mechanism controlling stress induced stomatal closure. *Plants* 8:9. 10.3390/plants8010009 30609843 PMC6359458

[B36] Santiago J. Rodrigues A. Saez A. Rubio S. Antoni R. Dupeux F. (2009). Modulation of drought resistance by the abscisic acid receptor PYL5 through inhibition of clade A PP2Cs. *Plant J*. 60 575–588. 10.1111/j.1365-313X.2009.03981.x 19624469

[B37] Sato A. Sato Y. Fukao Y. Fujiwara M. Umezawa T. Shinozaki K. (2009). Threonine at position 306 of the KAT1 potassium channel is essential for channel activity and is a target site for ABA-activated SnRK2/OST1/SnRK2.6 protein kinase. *Biochem. J.* 424 439–448. 10.1042/BJ20091221 19785574

[B38] Schroeder J. I. Allen G. J. Hugouvieux V. Kwak J. M. Waner D. (2001). Guard cell signal transduction. *Annu. Rev. Plant Physiol. Plant Mol. Biol.* 52 627–658. 11337411 10.1146/annurev.arplant.52.1.627

[B39] Schroeder J. I. Raschke K. Neher E. (1987). Voltage dependence of K^+^ channels in guard-cell protoplasts. *Proc. Natl. Acad. Sci. U.S.A.* 84 4108–4112. 10.1073/pnas.84.12.4108 16593851 PMC305032

[B40] Shimazaki K. I. Doi M. Assmann S. M. Kinoshita T. (2007). Light regulation of stomatal movement. *Annu. Rev. Plant Biol.* 58 219–247. 10.1146/annurev.arplant.57.032905.105434 17209798

[B41] Takemiya A. Kinoshita T. Asanuma M. Shimazaki K. (2006). Protein phosphatase 1 positively regulates stomatal opening in response to blue light in *Vicia faba*. *Proc. Natl. Acad. Sci. U.S.A.* 103 13549–13554. 10.1073/pnas.0602503103 16938884 PMC1569200

[B42] Takemiya A. Sugiyama N. Fujimoto H. Tsutsumi T. Yamauchi S. Hiyama A. (2013). Phosphorylation of BLUS1 kinase by phototropins is a primary step in stomatal opening. *Nat. Commun.* 4:2094. 10.1038/ncomms3094 23811955

[B43] Toda Y. Toh S. Bourdais G. Robatzek S. Maclean D. Kinoshita T. (2018). DeepStomata: Facial recognition technology for automated stomatal aperture measurement. *BioRxiv* [preprint]. 10.1101/365098

[B44] Toh S. Inoue S. Toda Y. Yuki T. Suzuki K. Hamamoto S. (2018). Identification and characterization of compounds that affect stomatal movements. *Plant Cell Physiol*. 59 1568–1580. 10.1093/pcp/pcy061 29635388

[B45] Tomiyama M. Inoue S. Tsuzuki T. Soda M. Morimoto S. Okigaki Y. (2014). Mg-chelatase I subunit 1 and Mg-Protoporphyrin IX methyltransferase affect the stomatal aperture in *Arabidopsis thaliana*. *J. Plant Res.* 127 553–563. 10.1007/s10265-014-0636-0 24840863 PMC4683165

[B46] Vahisalu T. Kollist H. Wang Y. F. Nishimura N. Chan W. Y. Valerio G. (2008). SLAC1 is required for plant guard cell S-type anion channel function in stomatal signalling. *Nature* 452 487–491. 10.1038/nature06608 18305484 PMC2858982

[B47] Yan N. Doelling J. H. Falbel T. G. Durski A. M. Vierstra R. D. (2000). The ubiquitin-specific protease family from *Arabidopsis*. *At*UBP1 and 2 are required for the resistance to the amino acid analog canavanine. *Plant Physiol*. 124 1828–1843. 10.1104/pp.124.4.1828 11115897 PMC59878

[B48] Ye W. Munemasa S. Shinya T. Wu W. Ma T. Lu J. (2020). Stomatal immunity against fungal invasion comprises not only chitin-induced stomatal closure but also chitosan-induced guard cell death. *Proc. Natl. Acad. Sci. U.S.A*. 117 20932–20942. 10.1073/pnas.1922319117 32778594 PMC7456093

[B49] Ye W. Murata Y. (2016). Microbe associated molecular pattern signaling in guard cells. *Front. Plant Sci*. 7:583. 10.3389/fpls.2016.00583 27200056 PMC4855242

[B50] Zhao J. Zhou H. Zhang M. Gao Y. Li L. Gao Y. (2016). Ubiquitin-specific protease 24 negatively regulates abscisic acid signalling in *Arabidopsis thaliana*. *Plant Cell Environ*. 39 427–440. 10.1111/pce.12628 26290265

[B51] Zhou H. Zhao J. Cai J. Patil S. B. (2017). UBIQUITIN-SPECIFIC PROTEASES function in plant development and stress responses. *Plant Mol. Biol.* 94 565–576. 10.1007/s11103-017-0633-5 28695315

