# Supplementary material for: Protease Inhibitor-Dependent Inhibition of Light-Induced Stomatal Opening
Source: Front Plant Sci. 2021 Sep 10;12:735328. doi: 10.3389/fpls.2021.735328 (PMC8462734; doi:10.3389/fpls.2021.735328)
Supplement: Supplementary file 1 [file Data_Sheet_1.pdf]

Figure S1

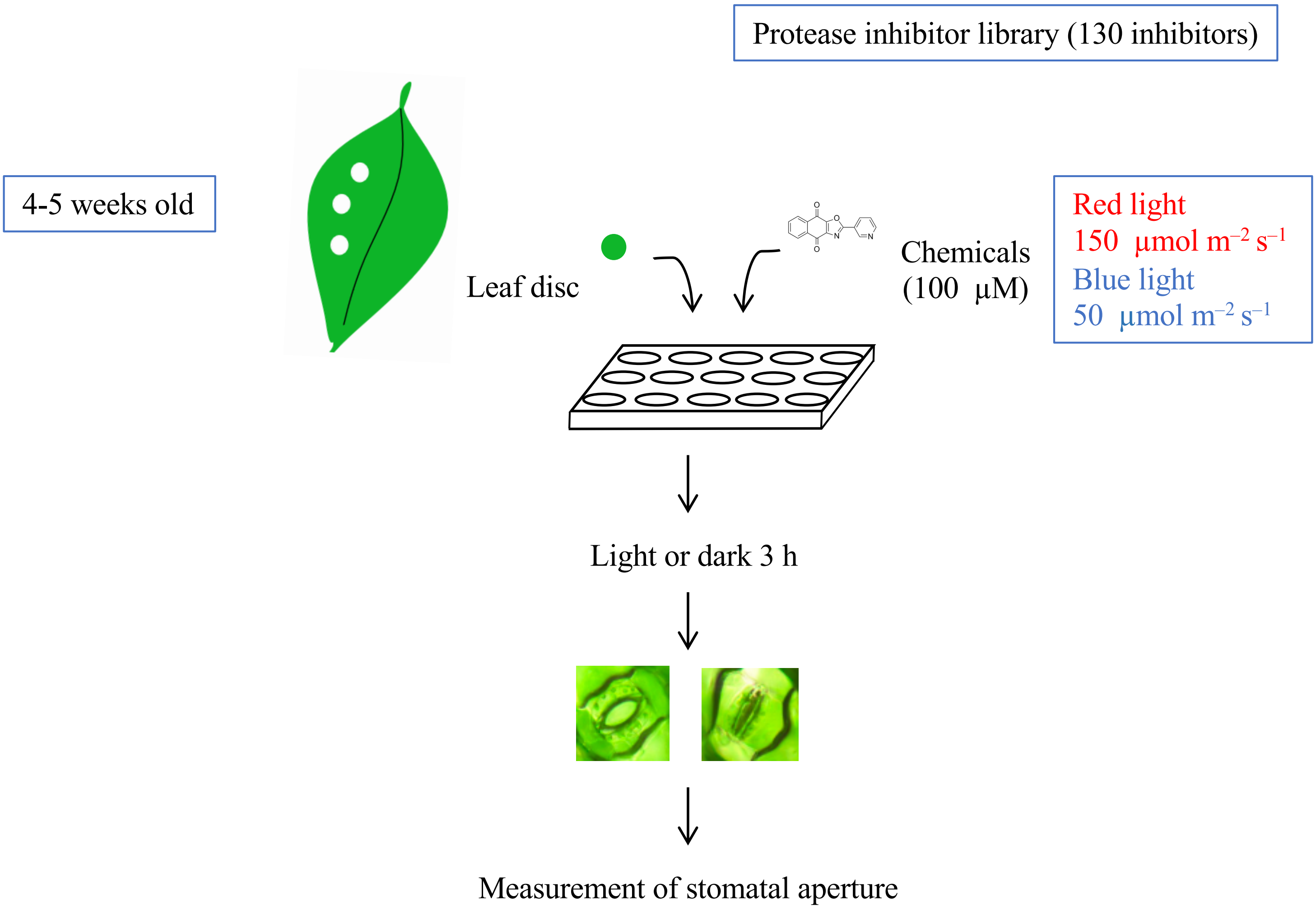

SUPPLEMENTARY FIGURE 1. Selection of compounds that affect stomatal movement. Leaf discs were isolated from *C. benghalensis* and incubated in basal reaction buffer with 100 µM inhibitors in multi-well plates. Stomatal aperture from abaxial side was measured by microscope after light or dark treatments for 3 h according to the previous methods (Ando and Kinoshita, 2018).
