# Supplementary material for: Protease Inhibitor-Dependent Inhibition of Light-Induced Stomatal Opening
Source: Front Plant Sci. 2021 Sep 10;12:735328. doi: 10.3389/fpls.2021.735328 (PMC8462734; doi:10.3389/fpls.2021.735328)
Supplement: Supplementary file 4 [file Data_Sheet_4.PDF]

Figure S4

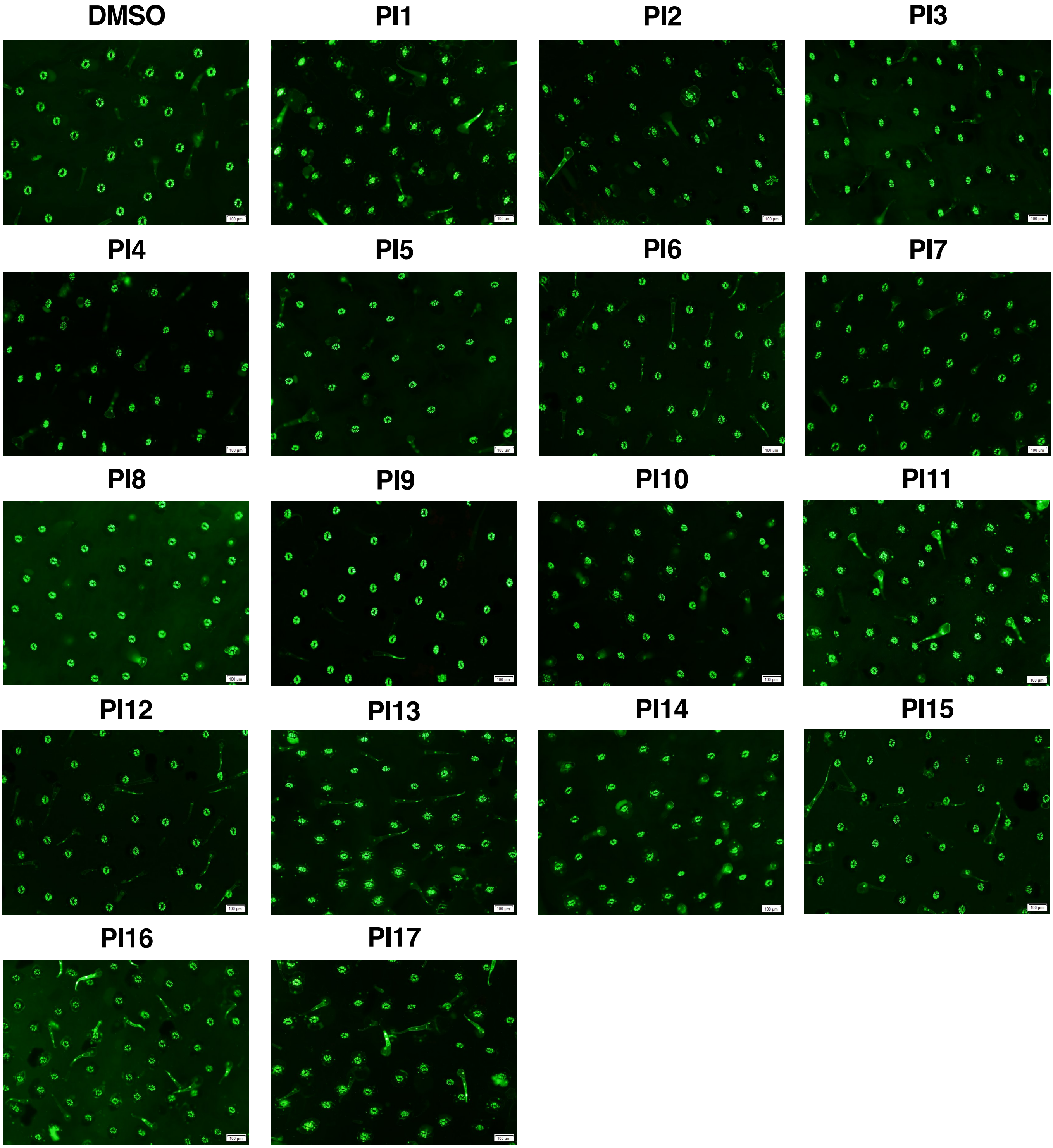

SUPPLEMENTARY FIGURE 4. Viability of guard cells from *C. benghalensis*. Leaf discs from *C. benghalensis* were treated with 100 μM PIs and illuminated with light (150 μmol m^{-2} s^{-1} red light and 50 μmol m^{-2} s^{-1} BL) for 3 h. The abaxial epidermis was removed from leaf discs and incubated in basal buffer containing 1 μg/mL FDA for 15 min. Fluorescence was detected using a fluorescence microscope. Bars represent 100 μm. Experiments repeated on three different occasions with similar results.
