# Supplementary material for: Protease Inhibitor-Dependent Inhibition of Light-Induced Stomatal Opening
Source: Front Plant Sci. 2021 Sep 10;12:735328. doi: 10.3389/fpls.2021.735328 (PMC8462734; doi:10.3389/fpls.2021.735328)
Supplement: Supplementary file 5 [file Data_Sheet_5.PDF]

A

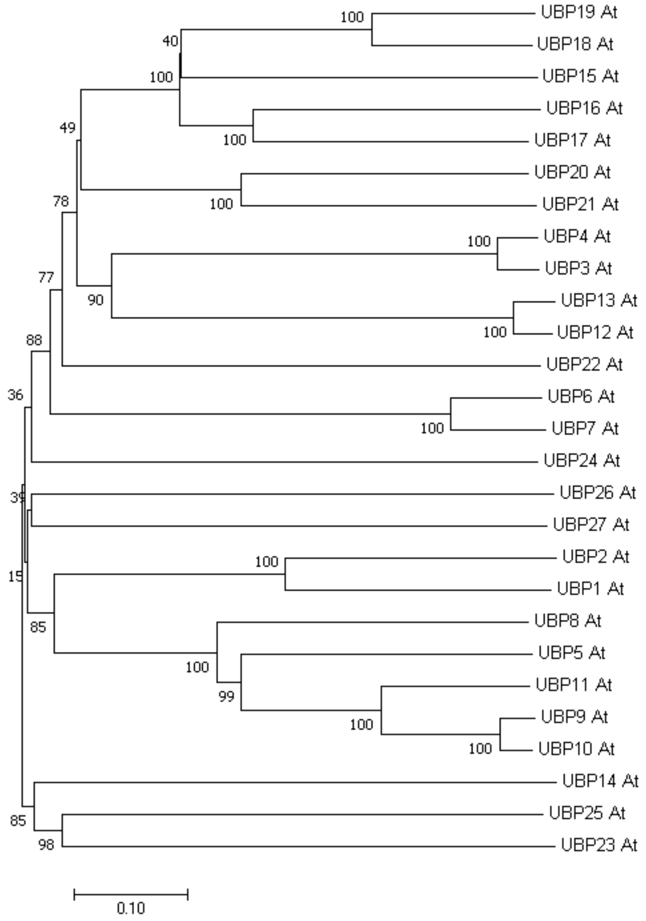

B

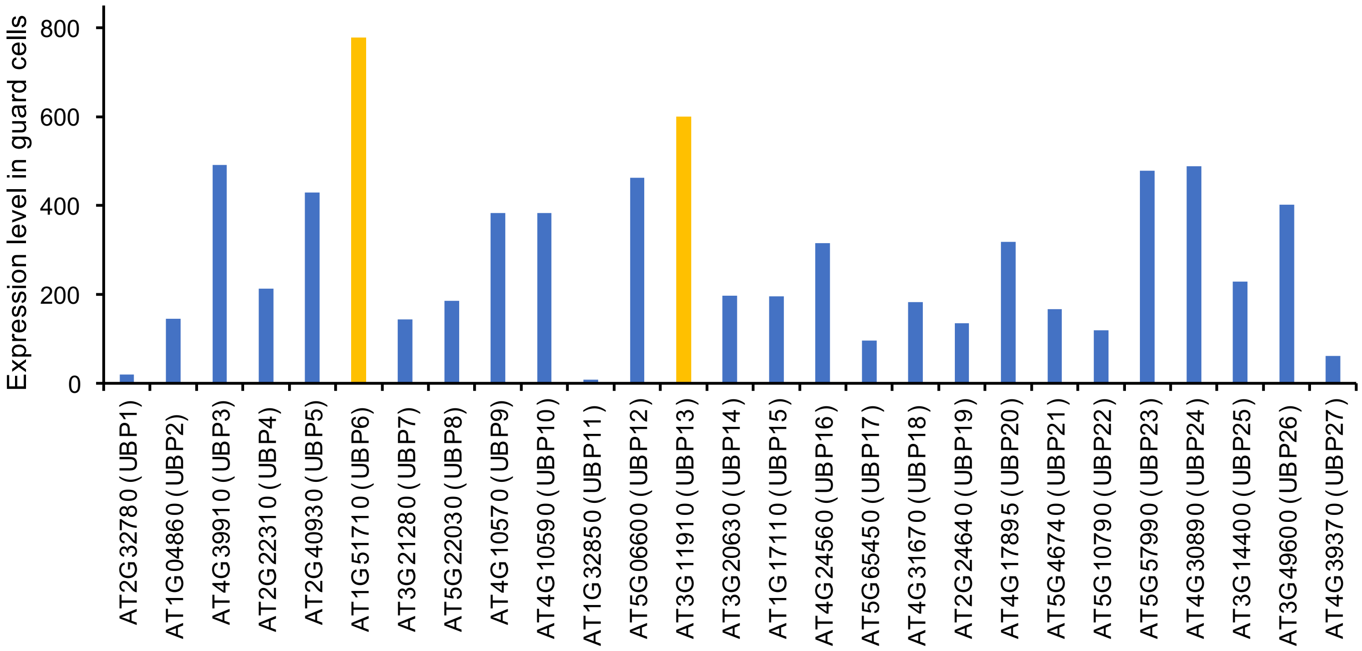

SUPPLEMENTARY FIGURE 5. Phylogenetic tree and expression levels of UBPs in guard cells of Arabidopsis. **(A)** Phylogenetic tree of 27 UBPs from Arabidopsis. Evolutionary analyses were conducted in MEGA 7.0 using the neighbour-joining method. Bootstrap tests using 1000 replicates are shown next to the branches. The evolutionary distances are in numbers of amino acid differences per site. **(B)** Expression levels of 27 UBPs in Arabidopsis. Data were obtained from Arabidopsis eFP Browser (http://bar.utoronto.ca/efp/cgi-bin/efpWeb.cgi?dataSource=Guard_Cell). Orange columns indicate UBPs expressed over 500 in expression level from guard cells of Arabidopsis.
