# Supplementary material for: Protease Inhibitor-Dependent Inhibition of Light-Induced Stomatal Opening
Source: Front Plant Sci. 2021 Sep 10;12:735328. doi: 10.3389/fpls.2021.735328 (PMC8462734; doi:10.3389/fpls.2021.735328)
Supplement: Supplementary file 6 [file Data_Sheet_6.pdf]

Figure S6

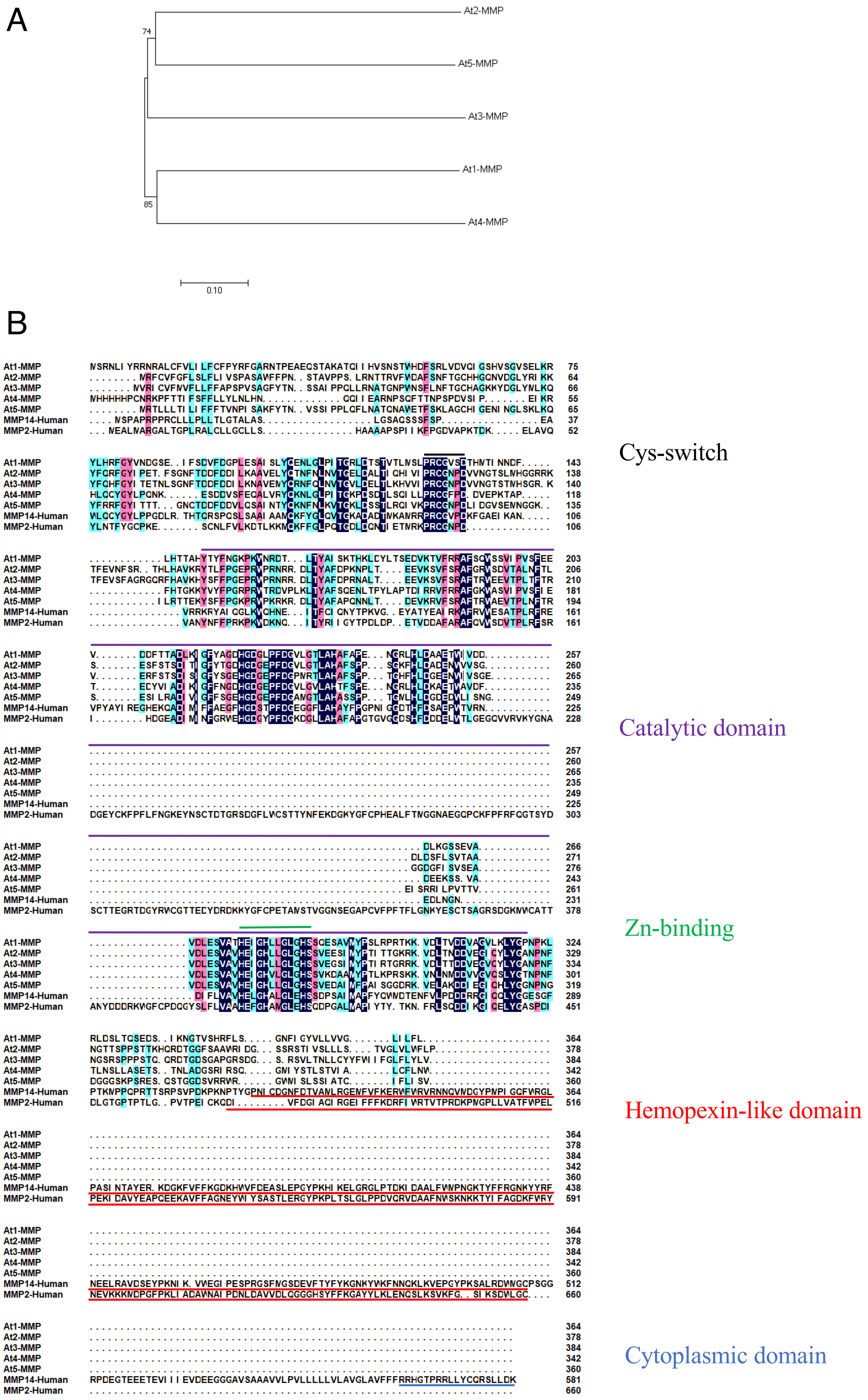
A
At2-MMP
At5-MMP
At3-MMP
At1-MMP
At4-MMP
74
85
0.10
B
Cys-switch
Catalytic domain
Zn-binding
Hemopexin-like domain
Cytoplasmic domain

C

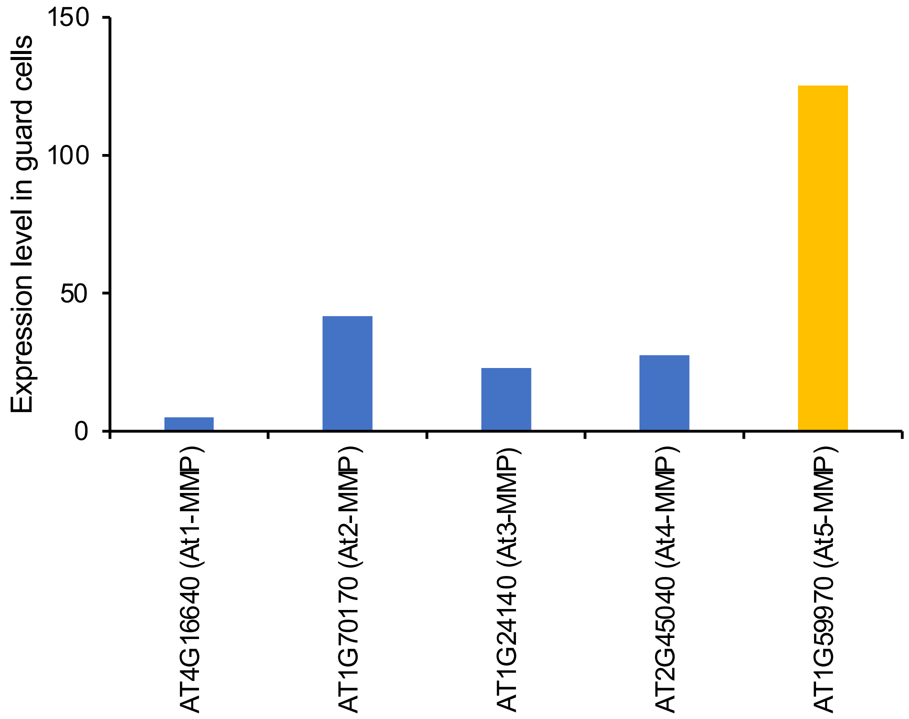

SUPPLEMENTARY FIGURE 6. Bioinformation of MMPs and their expression levels in guard cells of Arabidopsis. (A) Phylogenetic tree of MMPs from Arabidopsis. The tree was generated with MEGA7.0 using Neighbor-joining method. Bootstrap test from 1000 replicates were shown next to the branches. (B) Multiple sequence alignment of At-MMPs, MMP14 and MMP2 from human. The amino acid sequences of At-MMPs, MMP14 and MMP2 were extracted from Phytozome and NCBI database respectively. Alignment was performed by DNAman. Each domain was underlined with different colors (Liu *et al*., 2017; Liu *et al*., 2018). Hemopexin-like domain and cytoplasmic domain from human MMPs were determined using NCBI CD-search. The name of domains are shown on the right side. (C) Expression levels of 5 MMPs in Arabidopsis. Data were obtained from Arabidopsis eFP Browser (http://bar.utoronto.ca/efp/cgi-bin/efpWeb.cgi?dataSource=Guard_Cell).
