# Supplementary material for: Protease Inhibitor-Dependent Inhibition of Light-Induced Stomatal Opening
Source: Front Plant Sci. 2021 Sep 10;12:735328. doi: 10.3389/fpls.2021.735328 (PMC8462734; doi:10.3389/fpls.2021.735328)
Supplement: Supplementary file 7 [file Table_1.DOCX]

SUPPLEMENTARY TABLE 1. List of primers used in this study.

| Gene name | Forward (F)/Reverse (R) | Sequences (5’–3’) | Application |
| --- | --- | --- | --- |
| *RAB18* | F | TGTAACGCAGTCGCATTCG | quantitative RT-PCR |
|  | R | CACATCGCAGGACGTACATACAT |  |
| *RD29B* | F | CGAGCAAGACCCAGAAGTTCAC | quantitative RT-PCR |
|  | R | TTACCCGTTACACCACCTCTCA |  |
| *TUB2* | F | AAACTCACTACCCCCAGCTTTG | quantitative RT-PCR |
|  | R | CACCAGACATAGTAGCAGAAAT  CAAGT |  |
