# Supplementary material for: Protease Inhibitor-Dependent Inhibition of Light-Induced Stomatal Opening
Source: Front Plant Sci. 2021 Sep 10;12:735328. doi: 10.3389/fpls.2021.735328 (PMC8462734; doi:10.3389/fpls.2021.735328)
Supplement: Supplementary file 8 [file Table_2.docx]

SUPPLEMENTARY TABLE 2. Target of PIs in mammals.

|  | Name of inhibitor | Target in mammals |
| --- | --- | --- |
| PI1 | SJB3-019A | Ubiquitin-specific protease 1 (USP1) inhibitor |
| PI2 | NSC 405020 | Membrane type-1 matrix metalloproteinase inhibitor |
| PI3 | SB-3CT | Matrix metalloproteinase-2 (MMP-2) inhibitor |
| PI4 | MLN2238 | β5 site of the 20S proteasome inhibitor |
| PI5 | MLN9708 | β5 site of the 20S proteasome inhibitor |
| PI6 | Bortezomib (PS-341) | β5 site of the 20S proteasome inhibitor |
| PI7 | PI-1840 | Chymotrypsin-like proteasome inhibitor |
| PI8 | PAC-1 | Procaspase-3 activator |
| PI9 | BIBR-1048 | Thrombin inhibitor |
| PI10 | IPI-504 (Retaspimycin hydrochloride) | Hsp90 inhibitor |
| PI11 | Epoxomicin | 20S proteasome inhibitor |
| PI12 | HSP990 (NVP-HSP990) | Hsp90 inhibitor |
| PI13 | Semagacestat (LY450139) | γ-secretase inhibitor |
| PI14 | 17-AAG (KOS953) | Hsp90 inhibitor |
| PI15 | Flurizan | γ-secretase inhibitor |
| PI16 | E-64-c | Cysteine protease inhibitor |
| PI17 | Cathepsin G Inhibitor I | Cathepsin G inhibitor |

Information regarding target of PIs was from the PI library instruction.
