# Supplementary material for: Protease Inhibitor-Dependent Inhibition of Light-Induced Stomatal Opening
Source: Front Plant Sci. 2021 Sep 10;12:735328. doi: 10.3389/fpls.2021.735328 (PMC8462734; doi:10.3389/fpls.2021.735328)
Supplement: Supplementary file 9 [file Table_3.docx]

SUPPLEMENTARY TABLE 3. Putative targets of PI1 and PI3 in Arabidopsis.

| PIs | Mammalian target | Putative targets in Arabidopsis | Identity (%) |
| --- | --- | --- | --- |
| PI1 | USP1(Ubiquitin-specific protease1) | UBP3 (ubiquitin-specific protease 3), AT4G39910 | 33.71 |
|  |  | UBP4 (ubiquitin-specific protease 4), AT2G22310 | 33.33 |
|  |  |  |  |
| PI3 | Gelatinases A (matrix metalloproteinase-2, MMP2) | At5-MMP, AT1G59970 | 40.80 |
|  |  | At1-MMP, AT4G16640 | 40.74 |
|  |  | At3-MMP, AT1G24140 | 38.38 |
|  |  | At2-MMP, AT1G70170 | 37.85 |
|  |  | At4-MMP, AT2G45040 | 36.81 |
